# A preseason booster prolongs the increase of allergen specific IgG4 levels, after basic allergen intralymphatic immunotherapy, against grass pollen seasonal allergy

**DOI:** 10.1186/s13223-020-00427-z

**Published:** 2020-04-28

**Authors:** Dan Weinfeld, Ulla Westin, Laila Hellkvist, Ulf-Henrik Mellqvist, Ingvar Jacobsson, Lars-Olaf Cardell

**Affiliations:** 1 Asthma and Allergy Clinic Outpatient Unit (Adults), Department of Internal Medicine, South Alvsborgs Central Hospital, 50182 Boras, Sweden; 2 Division of Ear, Nose and Throat Diseases, Head and Neck Surgery, Department of Clinical Sciences, Lund University, Skane University Hospital, Lund, Sweden; 3 Department of ENT Diseases, Karolinska University Hospital, Stockholm, Sweden; 4 Section of Hematology, Department of Internal Medicine, South Alvsborgs Central Hospital, Boras, Sweden; 5 Clinical Chemistry, Department of Medical Imaging and Laboratory Medicine, South Alvsborgs Central Hospital, Boras, Sweden

**Keywords:** Intralymphatic immunotherapy, Randomized booster, IgG4, Grass pollen allergy rhinoconjunctivitis, Asthma

## Abstract

**Background:**

Allergen specific IgG4 levels have been monitored as a surrogate marker for the tolerance inducing effect of subcutaneous immunotherapy (SCIT) in many studies. Its accuracy at group level has been well established, but IgG4 has not yet found its place in the daily care of immunotherapy patients.

**Methods:**

Intralymphatic immunotherapy (ILIT) is a novel route for allergy vaccination against pollen allergy, where an ultrasound-guided injection of 1000 SQ-U Alutard is given directly into a groin lymph node. The suggested standard dosing so far has been one injection with 4 weeks in-between. In total 3000 SQ-U with the treatment completed in 2 months. IgG4 was measured with Immulite technique and rhinoconjunctivitis symptoms were estimated with daily online questionnaires. Mann–Whitney U-test and Wilcoxon Signed Rank test were applied for comparisons between groups and within groups, respectively.

**Results:**

The present study demonstrates that a single, preseason ILIT booster of 1000 SQ-U Alutard 5-grasses^®^, re-increases the allergen specific timothy-IgG4 levels, in patients already treated with ILIT before the previous pollen season. It also shows the feasibility of the ILIT-route for allergy vaccination of rhinitis patients, with or without concomitant asthma, with low degree of side effects and reconfirms high and sustained patient satisfaction.

**Conclusions:**

It is tempting to suggest that the allergen specific IgG4 levels can be used to build an intuitive algorithm for future clinical guidance of ILIT patients.

*Trial registration* Is Intralymphatic Allergen Immunotherapy Effective and Safe?, ClinicalTrials.gov Identifier NCT04210193. Registered 24 December 2019—Retrospectively registered, https://clinicaltrials.gov/ct2/show/study/NCT04210193?term=NCT04210193&draw=2&rank=1

## Background

Pollen induced allergic rhinoconjunctivitis is a worldwide heterogenic disease with underestimated morbidity. It is often accompanied by pollen induced asthma, or deterioration of chronic asthma [1]. The history of allergen specific immunotherapy (AIT) dates back more than 100 years [2, 3] and is the only disease modifying treatment that can induce long term symptom relief and decreased use of medication that remains several years after discontinuing the treatment [4]. Over decades, AIT practice patterns have diverged between Europe that prefers single aeroallergens with an adjuvant, like alum and the United States, where often allergen mixtures are given in aqueous solutions, without adjuvant [5]. Traditionally AIT is administered as subcutaneous injections (SCIT), repeated with increasing doses every week during an up-dosing phase of 2 to 3 months. The patients then continue to return for maintenance injections (100,000 SQ-U) every 6–8 weeks, during 3 years with aeroallergens and during 5 years with venom allergens. This makes SCIT time-consuming and somewhat inconvenient for the patients and costly for the healthcare providers. The alternative mode in routine care is sublingual immunotherapy (SLIT), where tablets or solutions are taken daily at home during at least 3 years. This approach is burdened by high cost of the medication and problems with compliance. In this context intralymphatic immunotherapy (ILIT) has emerged as an alternative form of AIT, both for pollen allergies [6–12] and indoor allergies [13–15] and was recently reviewed [16, 17]. In ILIT against pollen allergy, three injections with 4 weeks in-between, of low dose of allergen (1,000 SQ-U) are administrated to a lymph node in the groin. No dose finding ILIT-study has yet been published for pollen allergy, but escalated ILIT doses have been evaluated from a safety aspect both in individuals who previously were treated with 3 years of pollen SCIT- and in AIT-naive individuals [18]. Since the pioneer ILIT paper was published in 2008 [6], seven [6–8, 10–12, 19] out of eight published human ILIT trials, support the concept of ILIT against pollen allergies. Only one trial has been negative [9], and possible explanations have been debated [20]. ILIT’s efficacy appears to be similar to the effects induced by SCIT and SLIT, but with fewer side effects. However, despite some positive indications [6, 21, 22], we are still waiting for long termed, placebo controlled studies of the durability of this route of immunotherapy. Since the ILIT treatment period is relatively short, 2 months compared with at least 3 years for SCIT and SLIT, there has been a discussion about the potential beneficial effects of a single extra intralymphatic dose, given as a pre-seasonal booster. The problem with evaluating the booster effect is obvious, since the effect of any form of AIT, including ILIT is expected to last for several years. This makes it hard to evaluate the add-on effects, in real time, of a successful booster, without the use of a reliable biomarker.

Increase of allergen specific IgG4 has been suggested as a general maker of successful SCIT [23–27], even though its predictive value appears to be better at study group levels, than in individual cases. Hence, the present study was designed to evaluate changes in allergen specific IgG4 levels to timothy and control allergen birch, caused by a pre-seasonal booster given to patients previously treated with three intralymphatic injections of Alutard 5-grasses^®^.

## Methods

**Fig. 1:**
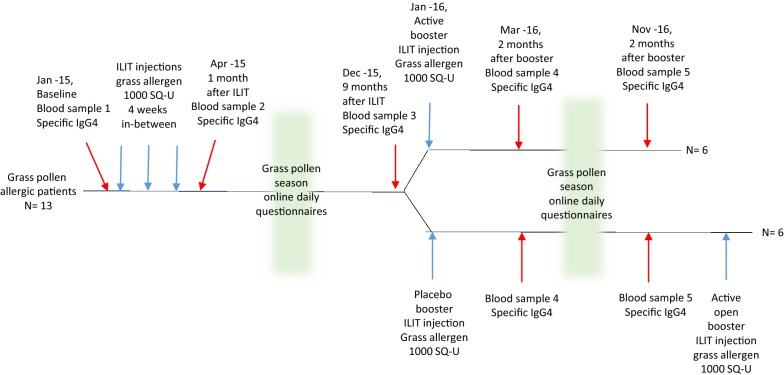
Grass allergic patients were treated with ILIT and then randomized double blind to an active or placebo ILIT booster dose after 1 year. Questionnaires were completed during the pollen seasons and blood samples for IgG4 measurements were obtained at several occasions

15 patients with a history of grass pollen induced allergic rhinitis confirmed by conjunctival grass allergen challenge, skin prick test and specific IgE (Immulite), were recruited during the autumn of 2014, from the SCIT waiting list. Two participants were lost due to pregnancy and moving, respectively. 13 participants were treated with three injections of 1000 SQ-U Alutard 5-grasses^®^ (ALK-Abello) with 4 weeks in-between. One woman became pregnant shortly after the 3d injection. In January 2016, the remaining 12 patients were double-blindly randomized by independent nurses with help of an online computer program, to receive either an intralymphatic booster injection of 1000 SQ-U ALK Alutard 5-grasses^®^ or placebo (given as ALK diluent^®^). (Additional file 1: Fig S1). The 12 patients were between 26 and 60 years old and nine of them had in addition to their grass pollen allergy, also allergy against birch pollen. Two patients had chronic asthma (stabilized on combined inhaler treatment) and at least 5 patients had grass pollen induced asthma that required inhaler treatment. The 6 patients who received placebo-booster in January 2016, got an active booster injection (4b) in December 2016, (after breaking the study code), equalizing the treatment doses (Fig. 1). The participants were allowed to medicate freely during the season with nasal steroids, antihistamines and eye-drops, according to their daily symptoms.

No patient hesitated to receive all 4 injections. All injections were performed inside an outpatient asthma and allergy clinic, operating at South Alvsborgs Central Hospital, without assistance from other departments. The unit is well equipped to handle anaphylactic reactions.

The study was approved by the ethics committee of Lund, registered with Clinical Trials gov ID NCT04210193, and was conducted according to good clinical practice. All participants signed a consent form.

A needle guide was used in combination with ultrasound (BK Medical Flex Focus 500) to direct the injections directly into the lymph node. All injections were performed by the corresponding author, assisted by the same two nurses, all with practical training from the ENT research unit at Karolinska University Hospital, Stockholm. All injections were recorded and saved on video. Examples of successful and failed injections are displayed online—(see Additional files 2, 3, 4, 5, 6, 7: Photo S1, Video S1a and S1b; Photo S2, Video S2a and S2b).

Re-examination of the films, showed that 75–80% of the injections were deposited completely inside the node, resulting in a transient nodal swelling, whereas 20–25% displayed signs of leakage into the surrounding tissues of the lymph node. With a few exceptions, the same lymph node was localized in each patient at every injection. The time consumed to deliver an injection, decreased during the study from 1 h to 10–20 min per injection. The technical differences of the depositions did not seem to influence the overall good safety profile of this treatment.

A titrated grass pollen skin prick test was performed at four occasions: At baseline, after the standard 3 first injections, before the booster injection and finally in the autumn 2016. Blood samples for allergen specific IgG4 (Immulite-technique, Siemens, expressed in μg/L) to timothy and birch measurements, were obtained at 5 occasions and stored frozen at minus 20 degrees: In January 2015 (baseline), in April 2015 (1 month after the 3d injection), in December 2015 (before randomized booster-injection, in January 2016), in March 2016 (approx. 2 months after booster injection), and in October–November 2016. In these 12 participants, all but one IgG4-sample has been included in the final analysis. One IgG4-sample was omitted due to a several months relapsing leg- erysipelas during the summer and autumn of 2016, preceding the last blood sampling.

10 of the 12 participants (6 with active booster and 4 with placebo booster), answered a daily online Google form questionnaire during the grass pollen seasons of 2015 and 2016 (37–41 days with about 75% response rate the same day, during the months of June and July, when the birch pollen season was terminated). The questionnaire scored the rhinoconjunctivitis symptoms (4 nasal and 3 ocular symptoms each ranging from 0 = no symptoms to 3 = severe symptoms), and use of medication, without dose specification.

Finally a repeated retrospective survey was sent out eleven times during the summers of 2015–2018, with 5 identical questions, asking the participants to estimate in a subjective manner, how they had responded to the treatment in general (if the injections had been effective, estimation of less or more symptoms, less or more use of medication and tiredness, compared to 2014 and before), and if they wanted to recommend ILIT to a friend.

### Statistical methods

Statistical inference was calculated, using the mean of two independent analyses of all timothy IgG4-measurements with a coefficient of variance between 5 and 9% (Additional file 8: Table S1). IgG4-measurements are presented as median and IQR and symptom scores as mean and SD. Symptom scores are calculated as a mean value for each participant over the 40 day period each summer. The Mann–Whitney U-test for continuous variables is used for comparisons between groups and the Wilcoxon Signed Rank test for comparisons within groups. The difference between IgG4 group medians are presented along with 95% confidence intervals using 100,000 replicates. Since the 95% CI:s are bootstrapped, they will not always correspond to the statistical testing p-values (Additional file 9: Table S2). Data were analyzed with SAS 9.4, SAS Institute Inc., Cary, NC, USA. A *p* value of 0.05 or less was considered statistically significant.

## Results

Altogether, 52 active injections of 1000 SQ ALK Alutard 5 grasses extract^®^ were given, without any acute systemic reactions. Leakage was more common from small lymph nodes, and they also tended to move with and away from the needle due to their lesser attachment area. Hence, nodes with a diameter of less than 6 mm, should be avoided. It was in nearly all cases possible to identify nodes bigger than 7 mm. The most common side effects reported were tiredness (19 injections, 36%) and mild nasal congestion at 24 h (6 injections, 11%). Transient local reactions at the site of injection were common and they consisted mainly of acute redness, swelling and itching. Generally, the 6 booster injections 2016 caused fewer local problems then the primary set of three injections 2015 (Table 1, Side-effects). No infections and no long term complications were seen. One patient developed breast malignancy, diagnosed 2½ years after the first injection, (6 months after injection 4b). At this point, no relationship with the injections is suspected.

**Table 1:** Side effects

	A	B	C	D	E	F
1	ILIT-injection × 3 basic treatment 2015 + booster-inj 2016	Basic ILIT treatment 1-3 and 39 injections	Active booster 13 injections	Placebo booster 6 injections		
2	Nose within 1 h	0	0	0		
3	Lungs within 1 h	0	0	0		
4	skin within 1 h	0	0	0		
5	Abdomen within 1 h	0	0	0		
6	Local redness within 1 h	1	1	0		
7	Local swelling within 1 h	4	4	0		
8	Local itch within 1 h	3	0	0		
9	Local bruising within 1 h	0	0	0		
10	Nose within 24 h	4	2	0		
11	Lungs within 24 h	0	0	0		
12	Skin within 24 h	3	1	0		
13	Abdomen within 24 h	0	0	0		
14	Tiredness within 24 h	16	3	1		
15	Local redness within 24 h	7	5	0		
16	Local swelling within 24 h	6	2	0		
17	Local within 24 h	9	4	0		
18	Local bruising within 24 h	0	0	0		
19						

The numbers indicate how many injections that caused the various side effects, respectively. In this project there were no local bruising

The basic treatment with three injections, from January to April 2015, caused a median increase of specific timothy IgG4 levels from 811 (IQR 287–1121) to 1937 (IQR 560–2183), resulting in a median increase of 686 (IQR 245–1338), p < 0.001 (n = 12). No such median increase was seen for birch IgG4 (the control allergen), that decreased slightly from 1451 (IQR 452–2835) to 1289 (IQR 453–2727), giving a median decrease of 130 (IQR − 552; − 37), p < 0,05 (n = 12).

**Fig. 2:**
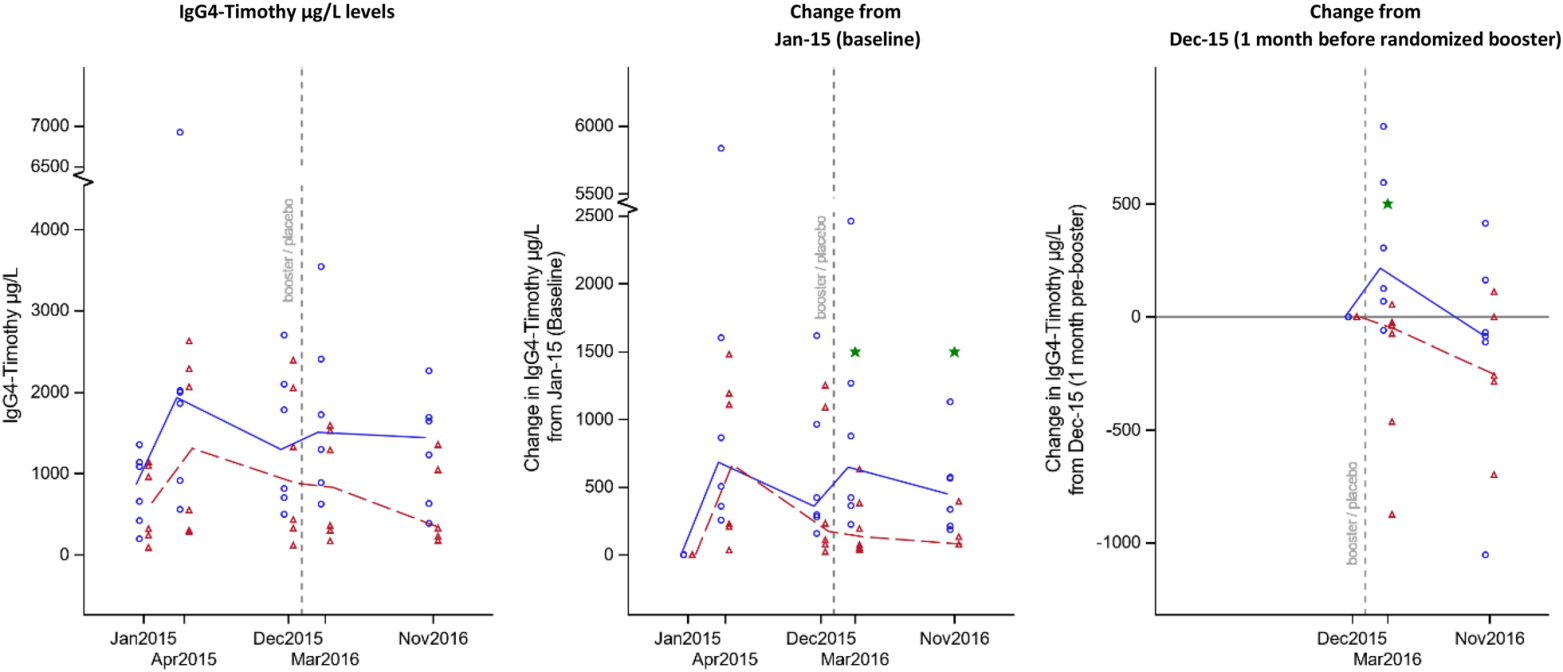
Specific IgG4-timothy by active booster (n = 6)/Placebo booster (n = 6) and changes from Jan-15 (baseline) and from Dec-15 (1 month pre-booster). All patients injected monthly 3 times from Jan 2015. Blue circles represent individuals who received a booster (4th) active injection Jan 2016. Red triangles received placebo (4th) injection in Jan 2016. Lines represents median values. * shows significant difference between active booster/placebo booster (p < 0.05)

The randomized active booster injection given in January 2016 caused an increase of the absolute allergen specific timothy—IgG4 levels, compared with previous year’s baseline, that was not seen in the placebo booster group. (March 2016: median increase 651; IQR 365–1272 and 137; IQR 57–383, respectively, n = 12, p < 0.05, median difference 514 (95% CI − 2; 1672)). This discrepancy remained at re-testing 8 months later (November 2016: median increase 451; IQR 214–575 and 81; IQR − 50 to + 134, respectively, n = 12, p < 0.05, median difference 370 (95% CI 10; 805)), (Fig. 2, middle graph). The outcome was similar when expressed as relative change **(**March 2016: median increase 160%; IQR 34–210% and 28%; IQR 18–66%, active versus placebo, respectively, n = 12, p < 0.05, median difference 132 percentage points (95% CI − 14; 194)), but the relative change did not remain significant in November 2016 (not shown in graphs, see Additional file 9: Table S2).

Calculating the change from December 2015 with reference date 1 month before the booster dose, (Fig. 2 right graph), the active booster increased the timothy IgG4 level significantly (March 2016: median increase 217; IQR 69–596 and median decrease 55; IQR − 462 to − 24, respectively, n = 12, p < 0.05, median difference 272 (95% CI 61; 1029)).

Expressed as relative change from December 2015, a non significant trend was found **(**March 2016: median increase 20%; IQR = 8–31% and median decrease 12%; IQR − 22 to − 3, active versus placebo, respectively, p = 0.066, median difference 32 percentage points (95% CI − 5; 71)).This difference was not statistically significant in November -2016, neither for absolute, nor for relative change.

All exact p-values and 95% CI:s are displayed in Additional file 9: Table S2.

The daily seasonal symptom evaluation (daily rhinoconjunctivitis score and symptom free days) did not reveal any significant difference between the group with active booster (n = 6) and placebo booster (n = 4, due to omitted scoring in 2 participants 2015) during the grass pollen season of 2016, in relation to the season of 2015. Neither did the skin prick test reactivity (with respect to histamine control) change.

However, within the active booster group, the total conjunctivitis symptom score decreased significantly during the summer of 2016 compared with 2015 (mean decrease 0.17; SD 0.09; n = 6, p < 0.05). The corresponding decrease was much smaller within the placebo booster group (mean decrease 0.06; SD 0.29; n = 4, p = 0.88), (Additional file 10: Table S3). A similar outcome for total conjunctivitis was obtained calculating the percentage symptom free days, that increased in the booster group (mean 14.3; SD 9.0; n = 6, p < 0.05), but not significantly in the placebo booster group (mean 4.6; SD 38.8, n = 4, p = 0.88), (Additional file 11: Table S4).

As for the 11 times repeated survey, answered during the summers of 2015–2018, the overall impression depicts a sustained patient satisfaction and a propensity to recommend this kind of treatment (Additional file 12: Table S5).

## Discussion

The present study demonstrates that a season booster of 1000 SQ-U prolongs the rise in allergen specific IgG4 to timothy. Even though an effect of total symptom reduction, with respect to the booster dose, could not be confirmed between groups in this small study, the total conjunctivitis mean symptom score decreased significantly within the active booster group. This was mirrored by a significant increase of eye symptom free days, within the booster group during the summer of 2016 compared to 2015. However, these results on eye symptoms came as a surprise, since the study was not powered to evaluate statistical significance for such parameters as symptoms or use of medication. The results might have been influenced of a slightly higher eye symptom score during the summer of 2015 in the group that would later receive active booster, compared to the group that would receive placebo booster.

Among the 13 participants, we note in particular 2 individuals that 5-double their IgG4-timothy level from baseline, after the 3 standard injections,(from 422 to 2024 and from 1086 to 6925 ng/L, respectively). Both displayed a clear re-rise in IgG4 after the active booster in Jan 2016 (and both claim very good clinical response with considerably less medication use). Three participants claimed no or only a marginal improvement in symptoms and these individuals all showed limited increase of their timothy specific IgG4-levels. In contrast one participant (the one who developed breast malignancy during 2017), reported a very good clinical response consistently during 2015–2018, but displayed no rise in Timothy-IgG4.

The systemic side effects noted were generally few and mild, dominated by tiredness and nasal congestion at 24 h post injection. The number of the latter appears presently to be a little more frequent than in previously reported studies [10], something that might be explained by a higher proportion of patients with asthma in this study. Asthmatic patients generally present allergen induced hyperreactivity both in the lower and in the upper airways. Reciprocally, patients with severe allergic rhinitis, often have a component of dyspnea and fulfil at least some diagnostic criteria of asthma. This might explain why some participants in this study reported late nasal symptoms. It is known from previous ILIT studies [14] that late systemic side effects, might signal a risk for anaphylactic reactions, at the next ILIT injection. It is therefore recommended that if dose increases are intended, (although not planned in this study), these should probably be avoided, if late systemic symptoms occur.

The theoretical reason for a beneficial role of high specific IgG4 levels in SCIT has been expanded by Aalberse et al. [28–33], notably the Fab arm exchange paradigm, that raises questions how to measure specific IgG4 and how a certain change in specific IgG4 during AIT, shall be interpreted. IgG4 is considered to mediate two main tolerance- inducing effects in AIT, first acting as a neutralizing antibody that competes with IgE for allergen binding and second, functioning as a blocking antibody, that prevents degranulation of effector cells. IgG4 also blocks IgE-facilitated binding of allergen- IgE-complexes to B-cells, a rate-limiting step for IgE-facilitated T-cell activation [34, 35]. IgG4 is the least abundant IgG subclass and accounts for less than 5% of total-IgG, but can reach up to 75% of total-IgG after chronic exposure to antigen, such as subcutaneous AIT. IgG4 might also have a damaging role in fibrotic inflammatory so called IgG4-related diseases and might impair antitumor response [36, 37]. Previous studies have confirmed that three intralymphatic injections of 1000 SQ-U of grass pollen with 4 weeks in-between, result in an increase of allergen specific IgG4, with a parallel reduction of the seasonal symptoms [12, 18]. Higher baseline timothy-IgG4 levels after completed previous SCIT, seem to facilitate a dose escalation protocol with intralymphatic grass allergen from 1000 SQ-U to 10,000 SQ-U during 2 months, which was not at all tolerated in a group of AIT-naive patients with grass pollen allergy [18]. Recently, IgG4 levels to wasp venom in patients with clonal mast cell disorders, who received venom immunotherapy, have been claimed to correlate with protection to field stings [38]. A paper investigating peanut oral immunotherapy under protection of omalizumab, observed that treatment success was preceded by a pronounced continued increase in IgG4 to whole peanut extract and to peanut components arah2 and arah6 [39].

The major advantages of ILIT over the current standard approved SCIT and SLIT routes, are the comparatively short treatment duration and the low allergen doses administered, which makes ILIT appeal both to patients and to healthcare givers. Even if the inconvenience factor is reduced substantially, there is still a need to identify prognostic biomarkers that can be utilized to assist patient selection, identification of responders and non-responders as well as detection of relapse and selection of patients who will benefit from a booster injection [23, 40]. As stated in the background-introduction, allergen specific IgG4 levels has been suggested as follow up marker during SCIT, to help determining the dose or change the vaccination protocol. In the present report, one single ILIT booster did cause a re-increase of the specific IgG4-level and it is tempting to speculate in the possibility that the booster may also increase the affinity to the allergen [7, 34, 41].

The argument that the affinity to the allergen does not correlate to the immunological level of IgG4, and therefore IgG4 cannot be used to monitor or steer immunotherapy in the individual patient, has been debated, as well as the concept of IgG4 being merely a surrogate biomarker reflecting other more general tolerance -inducing mechanisms [34, 42]. One could hypothesize that during certain circumstances, initiated B-cell tolerance with switch to specific IgG4 blocking antibodies, is not enough to reduce pollen-induced clinical symptoms. Explanations might be several -for instance that no parallel T-cell tolerance has been achieved, or perhaps that afferent sensory nerves, are still highly activated. In contrast to the slowly blunted IgE-response, specific IgG4 does usually not decrease, after an initial increase, as long as immunotherapy continues. It has also been suggested that functional serum and local nasal levels of specific IgG4 might be a better way to predict the individual clinical response [35, 43]. However, these sophisticated methods are laborious and costly and will probably not enter routine diagnostics in the near future. Another biomarker alternative in ASIT might be allergen specific IgA, probably more suitable for monitoring SLIT, but the data is so far limited [26, 27, 32, 44]. Early improvement in basophil sensitivity has been advocated to predict symptom relief with grass pollen immunotherapy [45]. A more cost efficient way with superior logistic aspects, of using BAT technology to monitor immunotherapy, might be so called BAT-inhibition [46, 47]. It can be noticed that the presently observed rise in IgG4-timothy after 3 ILIT-injections of 1000 SQ, in none of the 13 participants, reached IgG4-levels normally seen after 3 years of standard SCIT. The latter usually induces IgG4-levels of 50,000 ng/L or higher, in our clinic, which is comparable to the literature [32].

## Conclusion

To conclude, the presented allergen specific IgG4 data, together with the eye symptoms reported, indicate that the use of a pre-seasonal booster in conjunction with three basic injections of ILIT might be beneficial. It is enticing to speculate that further benefits from pre-seasonal boosters could be expected in terms of an increased duration of the treatment efficacy. However, the concept of a seasonal booster needs to be further developed, before it can be introduced into clinical praxis.

It may be easier, despite the considerable heterogeneity, to establish a clinical algorithm based on the specific high IgG4 dose response to ILIT, than for SCIT. The algorithm may include a combination of absolute and relative increase in specific IgG4. Based on our specific IgG4 levels that fluctuates in relation to tiny intralymphatic allergen injections, it is tempting to suggest that monitoring change of IgG4 have the potential to become a future tool, that can be used to decide the number of booster injections that an individual patient might need, to experience a sufficient and maintained clinical effect.

## Supplementary information

**Additional file 1: Figure S1.** Consort flow diagram.

**Additional file 2: Photo S1.** Two lymphatic nodes above the inguinal artery, in the left groin. The right nodule is 7 mm in diameter. The lymphatic core with micro vessels, verified by color doppler and surrounded by hypoechoic paracortical area (black).

**Additional file 3: Video S1a.** Shows a failed injection.

**Additional file 4: Video S1b.** Visualizes a successful injection.

**Additional file 5: Photo S2.** One larger lymphatic node, 10 mm in diameter with more prominent micro vessels, verified by color doppler.

**Additional file 6: Video S2a.** Shows a needle penetration of the capsule into the lymphatic tissue.

**Additional file 7: Video S2b.** illustrates an allergen injection into this lymph node, that is even more didactic, since the core of the lymph node is splitted for a short moment and simultaneously a transient swelling of the whole lymph node, is visualized.

**Additional file 8: Table S1.** CV% and other statistical measurements of IgG4-Timothy (ug/L). Double analyses 1 and 2 from 5 separate samplings (January 2015 until November 2016).

**Additional file 9: Table S2:** Specific IgG4 timothy by active booster/placebo booster (pregnant excluded).

**Additional file 10: Table S3:** Symptoms 2015 and 2016 and change in symptoms by booster Jan 2016.

**Additional file 11: Table S4:** Percent symptom free days 2015 and 2016 and change in percent symptom free days by booster Jan 2016 and symptom.

**Additional file 12: Table S5:** Survey, repeated 11 times during the grass pollen season 2015–2018.

## Data Availability

The datasets used and/or analyzed during the current study are available from the corresponding author upon reasonable request.
