# Supplementary material for: A preseason booster prolongs the increase of allergen specific IgG4 levels, after basic allergen intralymphatic immunotherapy, against grass pollen seasonal allergy
Source: Allergy Asthma Clin Immunol. 2020 Apr 28;16:31. doi: 10.1186/s13223-020-00427-z (PMC7189556; doi:10.1186/s13223-020-00427-z)
Supplement: Supplementary file 1 — Additional file 1: Figure S1. Consort flow diagram. [file 13223_2020_427_MOESM1_ESM.doc]

**
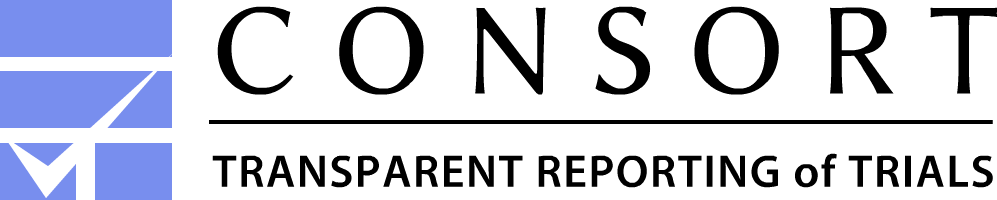
**

**CONSORT 2010 Flow Diagram**

**Allocation**

**Analysis**

**Follow-Up**

**Enrollment**

Assessed for eligibility (n=15)

Excluded (n= 1)

♦  Not meeting inclusion criteria (n= 0)

♦  Other reasons (n= 1 pregnancy)

Analysed (n=6)
♦ Excluded from analysis (give reasons) (n= 0)

Lost to follow-up (give reasons) (n=0)

Discontinued intervention (give reasons) (n= 0)

Allocated to intervention active booster (n= 6)

♦ Received allocated intervention (n=6)

♦ Did not receive allocated intervention (give reasons) (n= 0)

Lost to follow-up (give reasons) (n=0)

Discontinued intervention (give reasons) (n=0)

Allocated to intervention placebo booster (n= 6)

♦ Received allocated intervention (n= 6)

♦ Did not receive allocated intervention (give reasons) (n= 0)

Analysed (n=6)
♦ Excluded from analysis (give reasons) (n=0)

Randomized (n= 12)

Basic open label ILIT

3x1000 SQ-U (n= 13)

Excluded (n=2)

♦ Other reasons (n= 1 pregnancy,)

♦Declined to participate (n= 1 moving)
