# Supplementary figures and images for: A preseason booster prolongs the increase of allergen specific IgG4 levels, after basic allergen intralymphatic immunotherapy, against grass pollen seasonal allergy
Source: Allergy Asthma Clin Immunol. 2020 Apr 28;16:31. doi: 10.1186/s13223-020-00427-z (PMC7189556; doi:10.1186/s13223-020-00427-z)

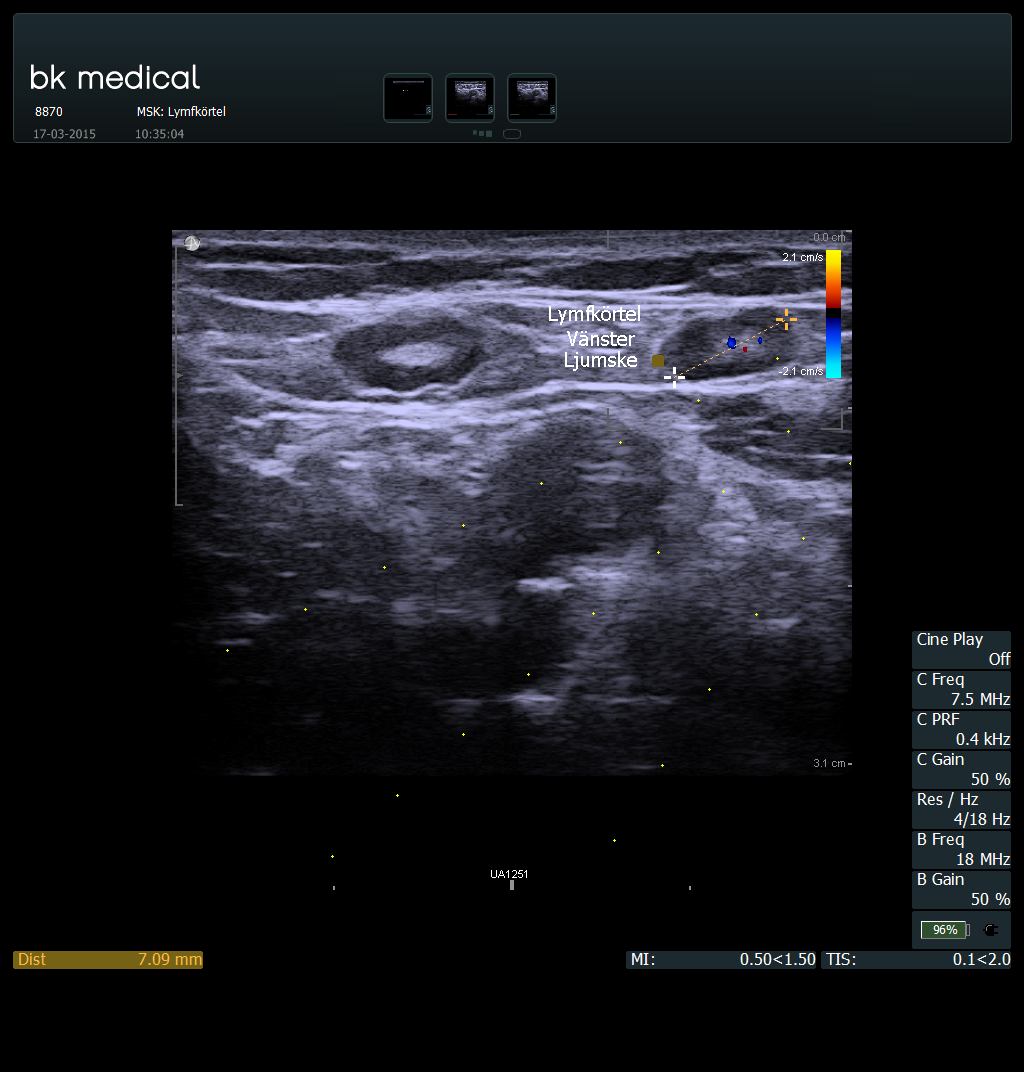

Supplement: Supplementary file 2 — Additional file 2: Photo S1. Two lymphatic nodes above the inguinal artery, in the left groin. The right nodule is 7 mm in diameter. The lymphatic core with micro vessels, verified by color doppler and surrounded by hypoechoic paracortical area (black). [file 13223_2020_427_MOESM2_ESM.bmp]

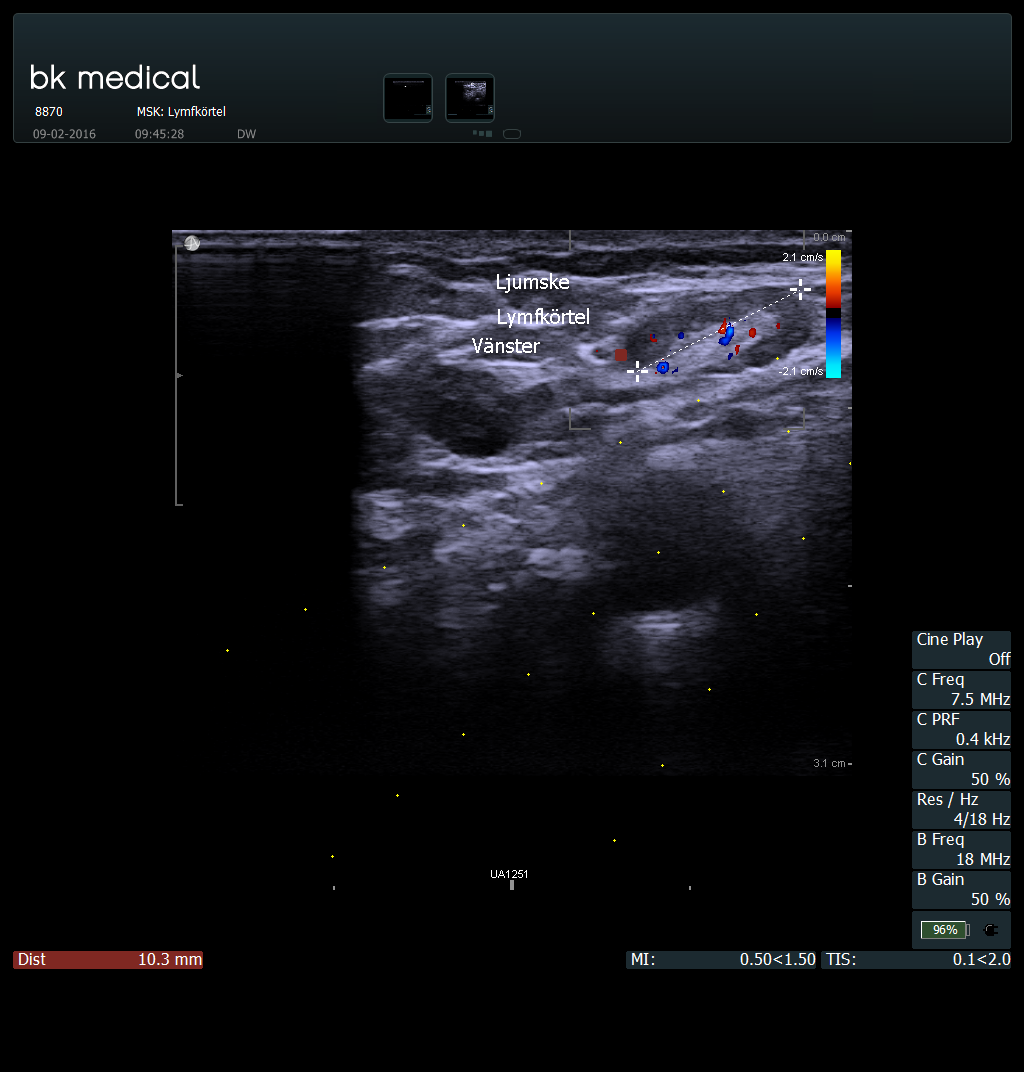

Supplement: Supplementary file 5 — Additional file 5: Photo S2. One larger lymphatic node, 10 mm in diameter with more prominent micro vessels, verified by color doppler. [file 13223_2020_427_MOESM5_ESM.bmp]
