# Supplementary material for: A preseason booster prolongs the increase of allergen specific IgG4 levels, after basic allergen intralymphatic immunotherapy, against grass pollen seasonal allergy
Source: Allergy Asthma Clin Immunol. 2020 Apr 28;16:31. doi: 10.1186/s13223-020-00427-z (PMC7189556; doi:10.1186/s13223-020-00427-z)
Supplement: Supplementary file 8 — Additional file 8: Table S1. CV% and other statistical measurements of IgG4-Timothy (ug/L). Double analyses 1 and 2 from 5 separate samplings (January 2015 until November 2016). [file 13223_2020_427_MOESM8_ESM.docx]

|  | **Difference Analysis 1 - Analysis 2** | |  | |
| --- | --- | --- | --- | --- |
| **Sampling Date** | **Mean (95% CI Limits of Agreement) (SD) Median (Min; Max) n=** | **Systematic changes p-value** | **CV %** | **Intra individual SD (IISD)** |
| Sampling 1 January - 2015 | 50.3 (-117.1; 217.7) (85.4) 25.0 (-42.0; 232.0) n=12 | 0.0640 | 9 | 67.90 |
| Sampling 2 April - 2015 | 49.9 (-181.4; 281.2) (118.0) 65.5 (-189.0; 193.0) n=12 | 0.1099 | 5 | 87.35 |
| Sampling 3 December - 2015 | 50.7 (-148.2; 249.5) (101.5) 31.5 (-113.0; 278.0) n=12 | 0.1563 | 6 | 77.47 |
| Sampling 4 March - 2016 | 78.5 (-54.7; 211.7) (68.0) 81.5 (3.0; 226.0) n=12 | 0.0005 | 5 | 72.10 |
| Sampling 5 Oct / Nov - 2016 | 32.9 (-88.0; 153.8) (61.7) 16.0 (-20.0; 206.0) n=11 | 0.0537 | 5 | 47.66 |
| CV is Coefficient of Variance (Intra individual SD * 100 / mean). Wilcoxon Signed Rank test is used to test the difference. For difference Mean (95% CI, Limits of Agreement) / (SD) / Median (Min; Max) / n= is presented. | | | | |
