# Supplementary material for: A preseason booster prolongs the increase of allergen specific IgG4 levels, after basic allergen intralymphatic immunotherapy, against grass pollen seasonal allergy
Source: Allergy Asthma Clin Immunol. 2020 Apr 28;16:31. doi: 10.1186/s13223-020-00427-z (PMC7189556; doi:10.1186/s13223-020-00427-z)
Supplement: Supplementary file 9 — Additional file 9: Table S2: Specific IgG4 timothy by active booster/placebo booster (pregnant excluded). [file 13223_2020_427_MOESM9_ESM.docx]

**Table S2 Specific IgG4 Timothy by Active booster/Placebo booster (pregnant excluded)**

|  | **Active booster ILIT grass allergen Alutard (sq) 1000+1000+1000+active booster 1000 sq (n=6)** | | | | | **Placebo booster ILIT grass allergen Alutard (sq) 1000+1000+1000+placebo booster 0 sq (n=6)** | | | | | **Difference of group medians**  **( 95% CI bootstrapped )**  **p-value** | | | |
| --- | --- | --- | --- | --- | --- | --- | --- | --- | --- | --- | --- | --- | --- | --- |
| **Blood sampling period** | **IgG4-Timothy (µg/L)** | **Change in IgG4-Timothy (µg/L) from Jan-15 (Baseline)** | **% change in IgG4-Timothy (µg/L) from Jan-15 (Baseline)** | **Change in IgG4-Timothy (µg/L) from Dec-15 (1 month pre-booster)** | **% change in IgG4-Timothy (µg/L) from Dec-15 (1 month pre-booster)** | **IgG4-Timothy (µg/L)** | **Change in IgG4-Timothy (µg/L) from Jan-15 (Baseline)** | **% change in IgG4-Timothy (µg/L) from Jan-15 (Baseline)** | **Change in IgG4-Timothy (µg/L) from Dec-15 (1 month pre-booster)** | **% change in IgG4-Timothy (µg/L) from Dec-15 (1 month pre-booster)** | **Change from Jan-15** | **% change from Jan-15** | **Change from Dec-15** | **% change from Dec-15** |
| **JAN2015** | 874 (422; 1141) |  |  |  |  | 644 (248; 1100) |  |  |  |  |  |  |  |  |
| **APR2015** | 1937 (920; 2024) | 686 (362; 1602) | 128 (39; 380) |  |  | 1314 (305; 2294) | 670 (210; 1194) | 112 (71; 129) |  |  |  |  |  |  |
| **DEC2015** | 1302 (704; 2106) | 360 (282; 965) | 76 (31; 147) |  |  | 886 (329; 2055) | 173 (81; 1094) | 34 (25; 109) |  |  |  |  |  |  |
| **MAR2016** | 1513 (889; 2413) | 651 (365; 1272) | 160 (34; 210) | 217 (69; 596) | 20 (8; 31) | 831 (305; 1531) | 137 (57; 383) | 28 (18; 66) | -55 (-462; -24) | -12 (-22; -3) | 514 (-2; 1672)  p 0.045 | 132 (-14; 194) p 0.045 | 272 (61; 1029)  p 0.020 | 32 (-5; 71) p 0.066 |
| **NOV2016** | 1444 (636; 1698) | 451 (214; 575) | 70 (51; 92) | -77 (-111; 165) | -7 (-22; 8) | 329 (229; 1050)  ^a^ | 81 (-50; 134)  ^a^ | 33 (-5; 41)  ^a^ | -257 (-283; 0)  ^a^ | -21 (-34; 0)  ^a^ | 370 (10; 805)  p 0.036 | 37  (-70; 114)  p 0.24 | 180 (-444; 629) p 0.52 | 14 (-99; 58) p 0.78 |
| IgG4 Timothy (µg/L) is presented with Median / (Q1; Q3)  ^a^ One IgG4 sample was omitted due to relapsing leg infection; n=5 instead of the total placebo booster group n=6 | | | | | | | | | | | | | | |
