# Supplementary material for: A preseason booster prolongs the increase of allergen specific IgG4 levels, after basic allergen intralymphatic immunotherapy, against grass pollen seasonal allergy
Source: Allergy Asthma Clin Immunol. 2020 Apr 28;16:31. doi: 10.1186/s13223-020-00427-z (PMC7189556; doi:10.1186/s13223-020-00427-z)
Supplement: Supplementary file 10 — Additional file 10: Table S3: Symptoms 2015 and 2016 and change in symptoms by booster Jan 2016. [file 13223_2020_427_MOESM10_ESM.docx]

**Table S3. Symptoms 2015 and 2016 and change in symptoms by booster jan 2016**

|  | **No booster (n=6)** | | **Booster jan 2016 (n=6)** | |  |
| --- | --- | --- | --- | --- | --- |
| **Variable** | **Mean (SD) Median (Min; Max) n=** | **p-value within group** | **Mean (SD) Median (Min; Max) n=** | **p-value within group** | **p-value between groups** |
| **2015** |  |  |  |  |  |
| **Itchy Nose** | 0.587 (0.431) 0.612 (0.125; 1.000) n=4 |  | 0.593 (0.607) 0.381 (0.000; 1.531) n=6 |  | 1.00 |
| **Runny Nose** | 0.742 (0.411) 0.828 (0.227; 1.085) n=4 |  | 0.441 (0.406) 0.359 (0.093; 1.166) n=6 |  | 0.24 |
| **Sneezings** | 0.610 (0.244) 0.516 (0.437; 0.971) n=4 |  | 0.477 (0.230) 0.570 (0.093; 0.694) n=6 |  | 1.00 |
| **Nose blockage** | 0.788 (0.572) 0.813 (0.090; 1.437) n=4 |  | 0.596 (0.448) 0.506 (0.071; 1.187) n=6 |  | 0.75 |
| **Rinitis Total symptoms** | 0.682 (0.305) 0.715 (0.284; 1.014) n=4 |  | 0.527 (0.382) 0.410 (0.125; 1.006) n=6 |  | 0.46 |
| **Red eyes** | 0.143 (0.115) 0.151 (0.000; 0.272) n=4 |  | 0.460 (0.230) 0.551 (0.000; 0.593) n=6 |  | 0.086 |
| **Itchy eyes** | 0.475 (0.351) 0.388 (0.156; 0.968) n=4 |  | 0.924 (0.241) 0.884 (0.607; 1.250) n=6 |  | 0.087 |
| **Tearing eyes** | 0.261 (0.045) 0.256 (0.218; 0.312) n=4 |  | 0.401 (0.282) 0.406 (0.071; 0.718) n=6 |  | 0.75 |
| **Conjunctivitis Total Symptoms** | 0.293 (0.150) 0.279 (0.125; 0.489) n=4 |  | 0.595 (0.112) 0.619 (0.404; 0.708) n=6 |  | 0.025 |
| **Combined Rhinitis Conjunctivitis Total** | 0.487 (0.161) 0.511 (0.278; 0.650) n=4 |  | 0.561 (0.207) 0.507 (0.354; 0.816) n=6 |  | 0.75 |
| **2016** |  |  |  |  |  |
| **Itchy Nose** | 0.304 (0.315) 0.230 (0.076; 0.925) n=6 |  | 0.450 (0.634) 0.231 (0.000; 1.703) n=6 |  | 1.00 |
| **Runny Nose** | 0.250 (0.435) 0.103 (0.000; 1.125) n=6 |  | 0.264 (0.389) 0.058 (0.000; 0.975) n=6 |  | 1.00 |
| **Sneezings** | 0.447 (0.319) 0.464 (0.000; 0.950) n=6 |  | 0.222 (0.223) 0.110 (0.035; 0.576) n=6 |  | 0.30 |
| **Nose Blockage** | 0.374 (0.476) 0.190 (0.000; 1.200) n=6 |  | 0.446 (0.447) 0.379 (0.000; 1.000) n=6 |  | 0.81 |
| **Rhinitis Total** | 0.344 (0.361) 0.214 (0.063; 1.050) n=6 |  | 0.345 (0.204) 0.362 (0.071; 0.575) n=6 |  | 0.47 |
| **Red Eyes** | 0.071 (0.103) 0.013 (0.000; 0.241) n=6 |  | 0.391 (0.244) 0.421 (0.000; 0.729) n=6 |  | 0.034 |
| **Itchy Eyes** | 0.339 (0.286) 0.321 (0.000; 0.763) n=6 |  | 0.662 (0.257) 0.610 (0.428; 1.148) n=6 |  | 0.093 |
| **Tearing Eyes** | 0.130 (0.173) 0.063 (0.000; 0.447) n=6 |  | 0.225 (0.174) 0.241 (0.025; 0.423) n=6 |  | 0.47 |
| **Conjunctivitis Total** | 0.180 (0.158) 0.165 (0.030; 0.456) n=6 |  | 0.426 (0.107) 0.424 (0.262; 0.540) n=6 |  | 0.031 |
| **Combined Rhinitis Conjunctivitis** | 0.262 (0.171) 0.241 (0.094; 0.542) n=6 |  | 0.386 (0.129) 0.427 (0.218; 0.533) n=6 |  | 0.23 |
| **2015 to 2016** |  |  |  |  |  |
| **Itchy Nose** | -0.216 (0.320) -0.062 (-0.696; -0.043) n=4 | 0.13 | -0.143 (0.430) -0.108 (-0.888; 0.336) n=6 | 0.63 | 0.75 |
| **Runny Nose** | -0.366 (0.477) -0.248 (-1.010; 0.040) n=4 | 0.25 | -0.177 (0.298) -0.134 (-0.562; 0.243) n=6 | 0.22 | 0.92 |
| **Sneezings** | -0.053 (0.159) -0.040 (-0.259; 0.127) n=4 | 0.63 | -0.256 (0.348) -0.341 (-0.619; 0.264) n=6 | 0.22 | 0.46 |
| **Nose Blockage** | -0.249 (0.411) -0.213 (-0.771; 0.200) n=4 | 0.38 | -0.150 (0.638) -0.034 (-1.187; 0.579) n=6 | 0.69 | 0.59 |
| **Rhinitis Total** | -0.221 (0.241) -0.198 (-0.525; 0.036) n=4 | 0.25 | -0.182 (0.342) -0.280 (-0.523; 0.294) n=6 | 0.31 | 0.92 |
| **Red Eyes** | -0.038 (0.145) -0.060 (-0.187; 0.157) n=4 | 0.63 | -0.069 (0.140) -0.058 (-0.263; 0.136) n=6 | 0.31 | 0.92 |
| **Itchy Eyes** | -0.089 (0.495) -0.254 (-0.457; 0.607) n=4 | 0.88 | -0.262 (0.180) -0.248 (-0.513; -0.089) n=6 | 0.031 | 0.75 |
| **Tearing Eyes** | -0.066 (0.222) -0.116 (-0.261; 0.229) n=4 | 0.63 | -0.176 (0.240) -0.189 (-0.533; 0.173) n=6 | 0.16 | 0.34 |
| **Conjunctivitis Total** | -0.064 (0.285) -0.148 (-0.293; 0.331) n=4 | 0.88 | -0.169 (0.093) -0.190 (-0.273; -0.003) n=6 | 0.031 | 1.00 |
| **Combined Rhinitis Conjunctivitis** | -0.285 (0.195) -0.205 (-0.576; -0.155) n=4 | 0.13 | -0.351 (0.424) -0.459 (-0.734; 0.291) n=6 | 0.16 | 0.59 |
| For continuous variables Mean (SD) / Median (Min; Max) / n= is presented. For comparison between groups the Mann-Whitney U-test was used for continuous variables. For comparison within groups the Wilcoxon Signed Rank test was used. | | | | | |
