# Supplementary material for: A preseason booster prolongs the increase of allergen specific IgG4 levels, after basic allergen intralymphatic immunotherapy, against grass pollen seasonal allergy
Source: Allergy Asthma Clin Immunol. 2020 Apr 28;16:31. doi: 10.1186/s13223-020-00427-z (PMC7189556; doi:10.1186/s13223-020-00427-z)
Supplement: Supplementary file 11 — Additional file 11: Table S4: Percent symptom free days 2015 and 2016 and change in percent symptom free days by booster Jan 2016 and symptom. [file 13223_2020_427_MOESM11_ESM.docx]

**Table S4. Percent symptom free days 2015 and 2016 and change in percent symptom free days by booster jan 2016 and symptom**

|  | **No booster (n=6)** | | **Booster jan 2016 (n=6)** | |  |
| --- | --- | --- | --- | --- | --- |
| **Variable** | **Mean (SD) Median (Min; Max) n=** | **p-value within group** | **Mean (SD) Median (Min; Max) n=** | **p-value within group** | **p-value between groups** |
| **2015** |  |  |  |  |  |
| **Itchy nose % 0 symptom** | 0.465 (0.372) 0.435 (0.114; 0.875) n=4 |  | 0.529 (0.425) 0.636 (0.000; 1.000) n=6 |  | 1.00 |
| **Runny nose % 0 symptom** | 0.357 (0.334) 0.313 (0.029; 0.773) n=4 |  | 0.629 (0.346) 0.722 (0.000; 0.906) n=6 |  | 0.24 |
| **Sneezings % 0 symptom** | 0.438 (0.198) 0.523 (0.143; 0.562) n=4 |  | 0.553 (0.218) 0.470 (0.361; 0.906) n=6 |  | 0.92 |
| **Nose blockage % 0 symptom** | 0.358 (0.413) 0.261 (0.000; 0.909) n=4 |  | 0.534 (0.335) 0.560 (0.000; 0.929) n=6 |  | 0.67 |
| **Rhinitis Total % 0 symptom** | 0.141 (0.241) 0.031 (0.000; 0.500) n=4 |  | 0.307 (0.274) 0.311 (0.000; 0.656) n=6 |  | 0.44 |
| **Red Eyes % 0 symptom** | 0.856 (0.116) 0.849 (0.727; 1.000) n=4 |  | 0.645 (0.184) 0.604 (0.464; 1.000) n=6 |  | 0.087 |
| **Itchy eyes % 0 symptom** | 0.594 (0.251) 0.641 (0.250; 0.844) n=4 |  | 0.318 (0.208) 0.369 (0.063; 0.594) n=6 |  | 0.11 |
| **Tearing eyes % 0 symptom** | 0.754 (0.028) 0.758 (0.719; 0.781) n=4 |  | 0.664 (0.255) 0.705 (0.281; 0.929) n=6 |  | 1.00 |
| **Conjunctivitis Total % 0 symptom** | 0.563 (0.237) 0.641 (0.219; 0.750) n=4 |  | 0.269 (0.203) 0.253 (0.031; 0.562) n=6 |  | 0.087 |
| **2016** |  |  |  |  |  |
| **Itchy nose % 0 symptom** | 0.720 (0.256) 0.770 (0.225; 0.923) n=6 |  | 0.693 (0.358) 0.769 (0.000; 1.000) n=6 |  | 1.00 |
| **Runny nose % 0 symptom** | 0.774 (0.374) 0.897 (0.025; 1.000) n=6 |  | 0.768 (0.377) 0.942 (0.025; 1.000) n=6 |  | 1.00 |
| **Sneezings % 0 symptom** | 0.590 (0.259) 0.536 (0.250; 1.000) n=6 |  | 0.815 (0.161) 0.890 (0.606; 0.964) n=6 |  | 0.13 |
| **Nose blockage % 0 symptom** | 0.663 (0.401) 0.810 (0.025; 1.000) n=6 |  | 0.579 (0.439) 0.698 (0.000; 1.000) n=6 |  | 0.69 |
| **Rhinitis Total % 0 symptom** | 0.419 (0.286) 0.393 (0.000; 0.750) n=6 |  | 0.306 (0.354) 0.241 (0.000; 0.857) n=6 |  | 0.81 |
| **Red Eyes % 0 symptom** | 0.929 (0.104) 0.988 (0.758; 1.000) n=6 |  | 0.691 (0.198) 0.685 (0.405; 1.000) n=6 |  | 0.051 |
| **Itchy Eyes % 0 symptom** | 0.670 (0.280) 0.679 (0.237; 1.000) n=6 |  | 0.469 (0.228) 0.528 (0.037; 0.654) n=6 |  | 0.30 |
| **Tearing Eyes % 0 symptom** | 0.870 (0.173) 0.937 (0.553; 1.000) n=6 |  | 0.817 (0.133) 0.792 (0.643; 0.975) n=6 |  | 0.47 |
| **Conjunctivitis Total % 0 symptom** | 0.657 (0.263) 0.679 (0.237; 0.925) n=6 |  | 0.412 (0.238) 0.420 (0.037; 0.654) n=6 |  | 0.23 |
| **2015 to 2016** |  |  |  |  |  |
| **Itchy nose % 0 symptom** | 0.201 (0.269) 0.080 (0.042; 0.602) n=4 | 0.13 | 0.164 (0.314) 0.109 (-0.183; 0.722) n=6 | 0.25 | 0.75 |
| **Runny nose % 0 symptom** | 0.305 (0.365) 0.216 (-0.004; 0.791) n=4 | 0.25 | 0.139 (0.226) 0.051 (-0.113; 0.469) n=6 | 0.22 | 0.59 |
| **Sneezings % 0 symptom** | 0.061 (0.133) 0.067 (-0.101; 0.213) n=4 | 0.38 | 0.262 (0.276) 0.322 (-0.104; 0.564) n=6 | 0.16 | 0.39 |
| **Nose blockage % 0 symptom** | 0.159 (0.179) 0.182 (-0.061; 0.333) n=4 | 0.25 | 0.045 (0.437) 0.020 (-0.579; 0.625) n=6 | 0.81 | 0.75 |
| **Rhinitis Total % 0 symptom** | 0.210 (0.151) 0.240 (0.000; 0.359) n=4 | 0.25 | -0.000 (0.357) 0.000 (-0.535; 0.557) n=6 | 1.00 | 0.24 |
| **Red Eyes % 0 symptom** | 0.038 (0.146) 0.060 (-0.158; 0.188) n=4 | 0.63 | 0.046 (0.143) 0.014 (-0.157; 0.263) n=6 | 0.44 | 0.92 |
| **Itchy Eyes % 0 symptom** | 0.033 (0.447) 0.170 (-0.607; 0.400) n=4 | 0.88 | 0.151 (0.128) 0.160 (-0.026; 0.342) n=6 | 0.063 | 0.92 |
| **Tearing Eyes % 0 symtom** | 0.051 (0.207) 0.101 (-0.228; 0.230) n=4 | 0.63 | 0.153 (0.211) 0.096 (-0.075; 0.534) n=6 | 0.094 | 0.67 |
| **Conjunctivitis Total % 0 symptom** | 0.046 (0.388) 0.185 (-0.513; 0.325) n=4 | 0.88 | 0.143 (0.090) 0.158 (0.006; 0.229) n=6 | 0.031 | 0.92 |
| For continuous variables Mean (SD) / Median (Min; Max) / n= is presented. For comparison between groups the Mann-Whitney U-test was used for continuous variables. For comparison within groups the Wilcoxon Signed Rank test was used. | | | | | |

**2018-12-19 Symptom.sas**
