# Supplementary material for: A preseason booster prolongs the increase of allergen specific IgG4 levels, after basic allergen intralymphatic immunotherapy, against grass pollen seasonal allergy
Source: Allergy Asthma Clin Immunol. 2020 Apr 28;16:31. doi: 10.1186/s13223-020-00427-z (PMC7189556; doi:10.1186/s13223-020-00427-z)
Supplement: Supplementary file 12 — Additional file 12: Table S5: Survey, repeated 11 times during the grass pollen season 2015–2018. [file 13223_2020_427_MOESM12_ESM.docx]

**Table S5, Survey, repeated 11 times during the grass pollen season 2015 - 2018**

| **Variable** | **June 2015 (n=12)** | **July 2015 (n=12)** | **June 2016 (n=10)** | **July 2016 (n=10)** | **August 2016 (n=10)** | **September 2016 (n=11)** | **July 2017 (n=9)** | **August 2017 (n=10)** | **May 2018 (n=11)** | **June 2018 (n=10)** | **July 2018 (n=8)** |
| --- | --- | --- | --- | --- | --- | --- | --- | --- | --- | --- | --- |
| **Subjective benefit of 3 +1 ILIT injections ?** |  |  |  |  |  |  |  |  |  |  |  |
| **Yes** | 8 (66.7%) | 9 (75.0%) | 6 (60.0%) | 6 (60.0%) | 7 (70.0%) | 7 (63.6%) | 8 (88.9%) | 7 (70.0%) | 9 (81.8%) | 8 (80.0%) | 6 (75.0%) |
| **Don’t know** | 4 (33.3%) | 2 (16.7%) | 2 (20.0%) | 2 (20.0%) | 2 (20.0%) | 2 (18.2%) | 1 (11.1%) | 2 (20.0%) | 1 (9.1%) | 2 (20.0%) | 1 (12.5%) |
| **No** | 0 (0.0%) | 1 (8.3%) | 2 (20.0%) | 2 (20.0%) | 1 (10.0%) | 2 (18.2%) | 0 (0.0%) | 1 (10.0%) | 1 (9.1%) | 0 (0.0%) | 1 (12.5%) |
| **Total symptom estimation** |  |  |  |  |  |  |  |  |  |  |  |
| **Much less symptoms** | 3 (27.3%) | 4 (33.3%) | 4 (40.0%) | 3 (30.0%) | 6 (60.0%) | 2 (18.2%) | 3 (33.3%) | 2 (20.0%) | 4 (36.4%) | 2 (20.0%) | 3 (37.5%) |
| **Less symptoms** | 6 (54.5%) | 5 (41.7%) | 2 (20.0%) | 5 (50.0%) | 3 (30.0%) | 7 (63.6%) | 4 (44.4%) | 6 (60.0%) | 6 (54.5%) | 6 (60.0%) | 4 (50.0%) |
| **No change in symptoms** | 1 (9.1%) | 2 (16.7%) | 3 (30.0%) | 2 (20.0%) | 1 (10.0%) | 2 (18.2%) | 2 (22.2%) | 2 (20.0%) | 1 (9.1%) | 2 (20.0%) | 1 (12.5%) |
| **More symptoms** | 1 (9.1%) | 1 (8.3%) | 1 (10.0%) | 0 (0.0%) | 0 (0.0%) | 0 (0.0%) | 0 (0.0%) | 0 (0.0%) | 0 (0.0%) | 0 (0.0%) | 0 (0.0%) |
| **Use of medication** |  |  |  |  |  |  |  |  |  |  |  |
| **Less use of medicines** | 9 (75.0%) | 7 (58.3%) | 6 (60.0%) | 6 (60.0%) | 7 (70.0%) | 8 (72.7%) | 6 (66.7%) | 6 (60.0%) | 6 (54.5%) | 6 (60.0%) | 5 (62.5%) |
| **No change** | 2 (16.7%) | 3 (25.0%) | 2 (20.0%) | 1 (10.0%) | 0 (0.0%) | 2 (18.2%) | 1 (11.1%) | 3 (30.0%) | 2 (18.2%) | 1 (10.0%) | 2 (25.0%) |
| **More use of medicines** | 1 (8.3%) | 2 (16.7%) | 2 (20.0%) | 3 (30.0%) | 3 (30.0%) | 1 (9.1%) | 2 (22.2%) | 1 (10.0%) | 3 (27.3%) | 3 (30.0%) | 1 (12.5%) |
| **Evaluation of tiredness** |  |  |  |  |  |  |  |  |  |  |  |
| **Less tired** | 7 (58.3%) | 8 (66.7%) | 3 (30.0%) | 5 (50.0%) | 6 (60.0%) | 5 (45.5%) | 5 (55.6%) | 6 (60.0%) | 6 (54.5%) | 6 (60.0%) | 3 (37.5%) |
| **No change** | 4 (33.3%) | 3 (25.0%) | 7 (70.0%) | 4 (40.0%) | 4 (40.0%) | 6 (54.5%) | 4 (44.4%) | 4 (40.0%) | 5 (45.5%) | 4 (40.0%) | 5 (62.5%) |
| **More tired** | 1 (8.3%) | 1 (8.3%) | 0 (0.0%) | 1 (10.0%) | 0 (0.0%) | 0 (0.0%) | 0 (0.0%) | 0 (0.0%) | 0 (0.0%) | 0 (0.0%) | 0 (0.0%) |
| **Recommendation ILIT to a friend** |  |  |  |  |  |  |  |  |  |  |  |
| **Yes** | 11 (91.7%) | 10 (83.3%) | 7 (70.0%) | 8 (80.0%) | 9 (90.0%) | 9 (81.8%) | 9 (100.0%) | 9 (90.0%) | 10 (90.9%) | 9 (90.0%) | 7 (87.5%) |
| **Don’t know** | 1 (8.3%) | 2 (16.7%) | 2 (20.0%) | 2 (20.0%) | 1 (10.0%) | 2 (18.2%) | 0 (0.0%) | 1 (10.0%) | 1 (9.1%) | 1 (10.0%) | 1 (12.5%) |
| **No** | 0 (0.0%) | 0 (0.0%) | 1 (10.0%) | 0 (0.0%) | 0 (0.0%) | 0 (0.0%) | 0 (0.0%) | 0 (0.0%) | 0 (0.0%) | 0 (0.0%) | 0 (0.0%) |
| For categorical variables n (%) is presented. | | | | | | | | | | | |
